# Association of dentoskeletal morphology with incisor inclination in angle class II patients: a retrospective cephalometric study

**DOI:** 10.1186/1746-160X-9-24

**Published:** 2013-09-03

**Authors:** Christian Kirschneck, Piero Römer, Peter Proff, Carsten Lippold

**Affiliations:** 1 Department of Orthodontics, University Medical Centre of Regensburg, Franz-Josef-Strauß-Allee 11, 93053 Regensburg, Germany; 2 Department of Orthodontics, University Medical Centre of Muenster, Waldeyerstraße 30, 48149 Münster, Germany

**Keywords:** Orthodontics, Cephalometry, Malocclusion, Angle Class II, Tooth inclination, Retrospective studies

## Abstract

**Introduction:**

The purpose of this study was to identify possible dentoskeletal parameters associated with variation of anterior tooth inclination in Angle Class II subdivisions.

**Methods:**

Pre-treatment lateral radiographs of 144 Class II patients (68 males, 76 females) aged 9 to 17 years were classified for upper incisor inclination into three groups (proclined, normally inclined, retroclined) homogeneous for gender and skeletal jaw relationship. The effect of age on the 22 cephalometric variables was controlled by covariance analysis.

**Results:**

Multivariate analysis of the cephalometric parameters indicated significant inter-group differences. Systematic associations with incisor inclination were revealed using rank correlation: Lower incisor proclination, Wits appraisal and gonial angle significantly decreased (0.04 ≥ p ≥ 0.002), while intercisal angle, mandibular total and corpus length and nasolabial angle increased (0.04 ≥ p ≥ 0.001) with decreasing incisor proclination.

**Conclusions:**

Clear-cut classification criteria and control of confounding effects may clarify conflicting previous findings on dentoskeletal differences between Class II subdivisions in the mixed dentition. Only minor dentoskeletal differences appear to be associated with incisor inclination. The increased interincisal and nasolabial angle in Class II division 2 subjects are due to reclination of both upper and lower incisors. Jaw positions and chin prominence are not significantly different between the subdivisions. However, Wits appraisal is decreased in Class II division 2. The increased mandibular length observed in Class II division 2 requires further scrutinization.

## Introduction

A thorough understanding of the dental and skeletal morphological features of specific malocclusions is pivotal to the selection of a causal therapeutic approach. The prevailing classification heuristics still rely upon *Angle*’s classic division based on dentoalveolar appearance. In consequence, the skeletal components constituting Class II division 1 and 2 malocclusions have to date remained vague. Related investigations often have only limited significance due to variable definition criteria, combining subdivisions, paucity of Class II division 2 cases and neglect of maturation effects on dentoskeletal morphology [1-3].

Recent studies statistically contrasting the skeletal features of the Class II subdivisions are scarce and have yielded conflicting results. Pancherz *et al.*[1] found no clear-cut skeletal differences besides incisor position between Class II divisions 1 and 2, while other authors have supported the existence of a clearly delimitable Class II division 2 morphology [2-5].

Upper incisor inclination is the paramount distinctive feature of Class II subdivisions. Anterior bite-deepening, on the other hand, is frequently included in the definition of a Class II division 2 malocclusion, while an open bite is no constitutive feature of Class II division 1. This definitional asymmetry may contribute to the inconsistent skeletal findings. Complexity may, therefore, be reduced using a subdivision uniquely based on labio-lingual inclination of the upper incisors [6].

This cephalometric study aimed to examine whether dentoskeletal features exist that systematically vary with upper incisor inclination in *Angle* Class II patients.

## Materials and methods

### Patients

The research was conducted in accordance with the declaration of Helsinki and the ethical regulations of the University of Regensburg, Germany.

The files of a private orthodontic practice were screened for pre-treatment cephalometric records of *Angle* Class II child and adolescent patients meeting the following criteria:

•bilateral distal occlusion in the anterior and posterior lateral segment with

≥ ¾ cusp width when the second lower primary molars were still in place

≥ ½ cusp width when the second primary molars had exfoliated to take the effect of the Leeway space into consideration;

•upper incisors fully erupted;

•no history of orthodontic treatment;

•no extractions of permanent teeth;

•absence of cleft deformities and syndromal craniofacial anomalies.

The angle between the upper central incisor axis and the nasal line (∠U1-NL) was used to distinguish for incisor inclination. Clinically, an angle of 110 degrees is considered normal, while inclination values falling below 106 degrees or exceeding 114 degrees indicate a significant deviation. Patients with normal values (within mean ± 1 standard deviation) according to corresponding population standards [7,8] and individualized norms (U1-NL_ind_ = 57.5 + 0.5 ML-NL; [9]) were assigned to the “normally inclined group” (II). Patients with elevated values were classified as “proclined” (group II/1), while values below average were classified as “retroclined” (group II/2).

Based on this classification of upper central incisor inclination, 144 patients were eligible for inclusion in this retrospective study:

•proclined (group II/1) n = 50 (34.7%),

•normally inclined (group II), n = 55 (38.2%)

•retroclined (group II/2) n = 39 (27.1%).

The patient sample included 76 females (52.8%) and 68 males (47.2%) equally distributed among the study groups (p = 0.67). The median age at the time of lateral cephalography was 11.5 years (mean 11.6 years, standard deviation 1.8 years) with an age range from 9 to 16.5 years (10% percentile 9.5 years, 90% percentile 14 years). The mean age of group II/2 was significantly higher (p < 0.001) compared with groups II and II/1 (Table 1).

**Table 1:** Age distribution among the test groups

		**Group**			**Total**
		**II/1 (n = 50)**	**II (n = 55)**	**II/2 (n = 39)**	
Age	Mean	10.8	11.5	12.6	11.6
	Median	10.5	11.5	12.5	11.5
	Standard deviation	1.5	1.6	1.8	1.8
	Minimum - maximum	9.0 – 14.5	9.0 – 15.5	9.5 – 16.5	9.0 – 16.5

### Cephalometric analysis

The lateral radiographs were analyzed by the same observer using a modified *Bergen/Hasund* analysis [10,11].

Measurements were obtained for 23 cephalometric parameters:

•upper/lower central incisor inclination (∠U1-NL, ∠L1-ML, ∠L1-NB, ∕L1-NB, ∠U1-L1);

•sagittal parameters (∠SNA, ∠SNB, ∠ANB, ∠SNPg, ∠PgNB, ∕Wits, mandibular total (∕Go-Pg) and corpus (∕Go-Gn) lengths, maxillary length (∕A’-PNS);

•vertical parameters (∠ML-NSL, ∠NL-NSL, ∠ML-NL, ∠ArGoMe, ramus length (∕Ar-Go), F(acial) H(eight) index (∕N-ANS : ∕ANS-Gn), *Jarabak*’s ratio (∕S-Go : ∕N-Me);

•soft tissue parameters (∠nasolabial angle, ∠H(*oldaway*)-angle)

**Figure 1:**
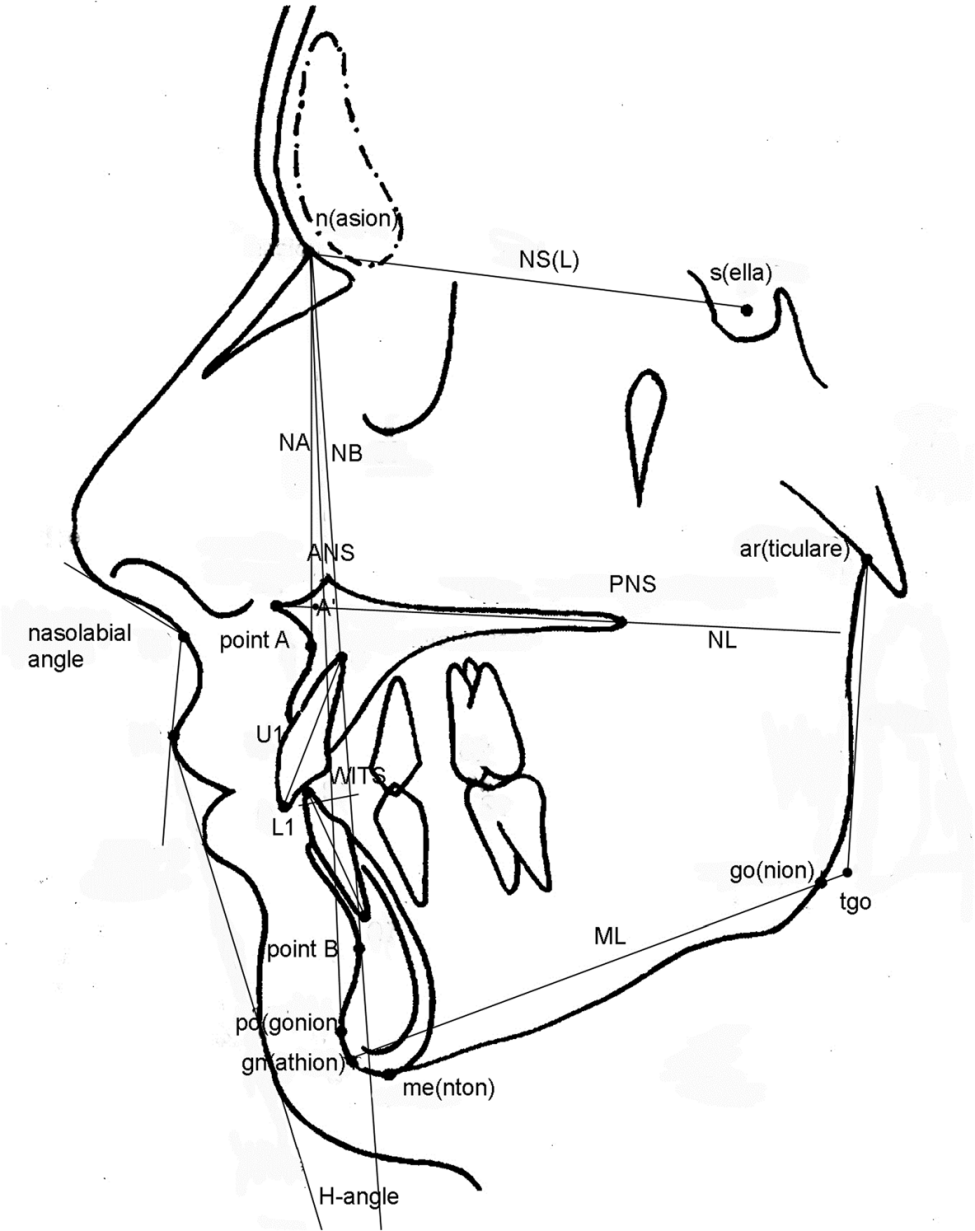
**Skeletal landmarks and soft tissue parameters: upper/lower central incisor inclination (**∠**U1-NL,** ∠**L1-ML,** ∠**L1-NB, ∕L1-NB,** ∠**U1-L1); sagittal parameters (**∠**SNA,** ∠**SNB,** ∠**ANB,** ∠**SNPg,** ∠**PgNB, ∕Wits, mandibular total (∕Go-Pg) and corpus (∕Go-Gn) lengths, maxillary length (∕A’-PNS); vertical parameters (**∠**ML-NSL,** ∠**NL-NSL,** ∠**ML-NL,** ∠**ArGoMe, ramus length (∕Ar-Go), F(acial) H(eight) index (∕N-ANS: ∕ANS-Gn), *Jarabak*’s ratio (∕S-Go : ∕N-Me); soft tissue parameters (**∠**nasolabial angle,** ∠***H*(*oldaway*)-angle).**

Angular measurements (∠) were in degrees (°), linear measurements in mm. The related skeletal landmarks and soft tissue parameters are shown in Figure 1.

In order to evaluate measurement error, the digitized cephalograms of 30 randomly selected patients were re-traced by the same investigator after 2 weeks. The error of method was calculated using the formula $\sqrt{\frac{\sum d^{2}}{2n}}$ where d is the difference between duplicated measurements and n the number of double measurements [12].

Random errors ranged from 0.34 mm to 0.67 mm for linear measurements, from 0.3 to 0.95 degrees for the angular measurements and from 0.55 to 0.85 per cent for proportions.

### Statistical data analysis

A multiple analysis of variance (MANOVA) revealed significant interactions of test group and patient age, while patient gender and presence of a distobasal relationship evaluated at cut-off values of the individualized ANB angle [13] and Wits appraisal [14]) were not significantly confounded with group membership. Since patient age was significantly different between groups, analysis of covariance (ANCOVA) controlling for patient age [15-17] was used to compare the cephalometric parameters between inclination groups. After the variance due to age was removed from the overall effect, the parameter means were adjusted by the method of least squares to the grand mean and tested after *Sokal* and *Rohlf*[18] for intergroup differences.

In order to reveal rank order associations of cephalometric parameters with incisor inclination, *Kendall*’s correlation coefficient τ was calculated between group membership (ordinally ranked by incisor inclination: II/1 – II – II/2) and the age-adjusted residual values (observed value minus value predicted by age).

The level of significance was established at p < 0.05. Analyses were run using the Statistical Package for Social Sciences, version 13 (SPSS Inc., Chicago, Illinois, USA).

## Results

The crude means and the age-adjusted linear regression-estimated means of the cephalometric parameters are given in Tables 2 and 3. Using analysis of variance, significant differences between groups were observed. Additional correlational analyses between upper incisor inclination and cephalometric parameters revealed significant systematic associations. The results of significance testing are presented in Table 4.

**Table 2:** Observed crude group means with S(tandard) E(rror of) M(ean)

	**Group II/1 (n = 50)**		**Group II (n = 55)**		**Group II/2 (n = 39)**	
	***Mean***	***SEM***	***Mean***	***SEM***	***Mean***	***SEM***
L1-ML (°)	97.6	0.8	95.8	0.9	94.5	1.0
L1-NB (°)	24.7	0.7	23.9	0.8	20.5	1.0
L1-NB (mm)	3.9	0.3	3.8	0.3	2.3	0.4
U1-L1 (°)	117.9	0.7	127.5	1.1	144.5	1.7
SNA (°)	79.9	0.5	80.7	0.6	80.7	0.6
SNB (°)	75.8	0.4	75.7	0.5	76.6	0.5
ANB (°)	4.2	0.3	5.0	0.3	4.2	0.3
Wits (mm)	3.1	0.4	3.0	0.4	1.7	0.4
SNPg (°)	77.1	0.4	76.7	0.5	78.1	0.6
PgNB (°)	2.6	0.2	2.0	0.3	3.0	0.3
Maxillary length (mm)	52.2	0.6	52.9	0.5	52.6	0.6
Mandibular length (mm)	77.5	0.7	79.2	0.7	82.0	0.9
Corpus length (mm)	71.8	0.7	73.2	0.7	76.3	0.8
Ramus length (mm)	57.1	0.6	57.3	0.7	57.1	1.1
ML-NSL (°)	31.2	0.6	32.4	0.9	29.5	0.9
NL-NSL (°)	7.9	0.4	7.3	0.5	8.1	0.6
ML-NL (°)	23.4	0.6	25.1	0.9	21.4	0.9
ArGoMe (°)	124.1	1.0	124.6	0.9	121.1	1.0
FH index (x100)	85.7	1.1	84.3	1.0	86.8	1.4
Jarabak’s ratio (x100)	66.3	0.5	65.8	0.7	67.9	0.9
H-angle (°)	13.7	0.6	13.9	0.6	11.1	0.8
Nasolabial angle	109.8	1.8	112.5	1.9	114.6	1.8

**Table 3:** Estimated age-adjusted marginal means with S(tandard) E(rror of) M(ean) broken down by test groups

	**Group II/1 (n = 50)**		**Group II (n = 55)**		**Group II/2 (n = 39)**	
	**Mean**	**SEM**	**Mean**	**SEM**	**Mean**	**SEM**
L1-ML (°)	97.7	0.9	95.8	0.8	94.4	1.1
L1-NB (°)	24.6	0.8	23.7	0.7	20.7	0.9
L1-NB (mm)	3.9	0.3	3.8	0.3	2.3	0.4
U1-L1 (°)	118.4	1.2	127.6	1.1	143.6	1.4
SNA (°)	79.9	0.6	80.7	0.6	80.7	0.7
SNB (°)	75.9	0.5	75.8	0.4	76.3	0.6
ANB (°)	4.1	0.3	4.9	0.3	4.4	0.4
Wits (mm)	3.2	0.4	3.0	0.4	1.5	0.5
SNPg (°)	77.3	0.5	76.8	0.5	77.7	0.6
PgNB (°)	2.7	0.3	2.1	0.2	2.8	0.3
Maxillary length (mm)	52.6	0.5	53.0	0.5	51.8	0.6
Mandibular length (mm)	78.1	0.8	79.0	0.7	81.0	0.9
Corpus length (mm)	72.3	0.7	73.1	0.7	75.4	0.8
Ramus length (mm)	57.9	0.7	57.5	0.7	55.7	0.8
ML-NSL (°)	31.0	0.8	32.3	0.8	30.0	1.0
NL-NSL (°)	7.9	0.5	7.3	0.5	8.0	0.6
ML-NL (°)	22.9	0.8	25.1	0.8	22.1	1.0
ArGoMe (°)	124.1	1.0	124.2	0.9	121.3	1.1
FH index (x100)	85.7	1.1	84.3	1.0	86.8	1.3
Jarabak’s ratio (x100)	66.7	0.7	65.9	0.6	67.2	0.8
H-angle (°)	13.5	0.7	13.8	0.6	11.6	0.8
Nasolabial angle	110.2	1.9	112.5	1.6	114.0	2.2

**Table 4:** Comparison of test groups adjusted for age: ANCOVA and*Kendall*’s*τ*;p* significant effects

	**ANCOVA**	**Rank correlation**	
	***p***	***τ***	***p***
L1-ML (°)	0.07	−0.19	0.01*
L1-NB (°)	0.01*	−0.21	0.005*
L1-NB (mm)	0.003*	−0.24	0.002*
∠U1-L1	0.001*	0.75	0.001*
SNA (°)	0.63	0.07	0.42
SNB (°)	0.77	0.03	0.70
ANB (°)	0.16	0.07	0.23
Wits (mm)	0.02*	−0.21	0.01*
SNPg (°)	0.47	0.03	0.75
PgNB (°)	0.11	0.03	0.62
Maxillary length (mm)	0.31	−0.07	0.29
Mandibular length (mm)	0.04*	0.23	0.001*
Corpus length (mm)	0.03*	0.25	0.001*
Ramus length (mm)	0.11	−0.12	0.05
ML-NSL (°)	0.17	−0.07	0.21
NL-NSL (°)	0.61	0.01	0.84
ML-NL (°)	0.05	−0.08	0.17
ArGoMe (°)	0.07	−0.14	0.04*
FH index	0.32	0.07	0.28
*Jarabak*’s ratio	0.42	0.05	0.37
*H*-angle (°)	0.08	−0.10	0.12
Nasolabial angle (°)	0.19	0.15	0.04*

**Figure 2:**
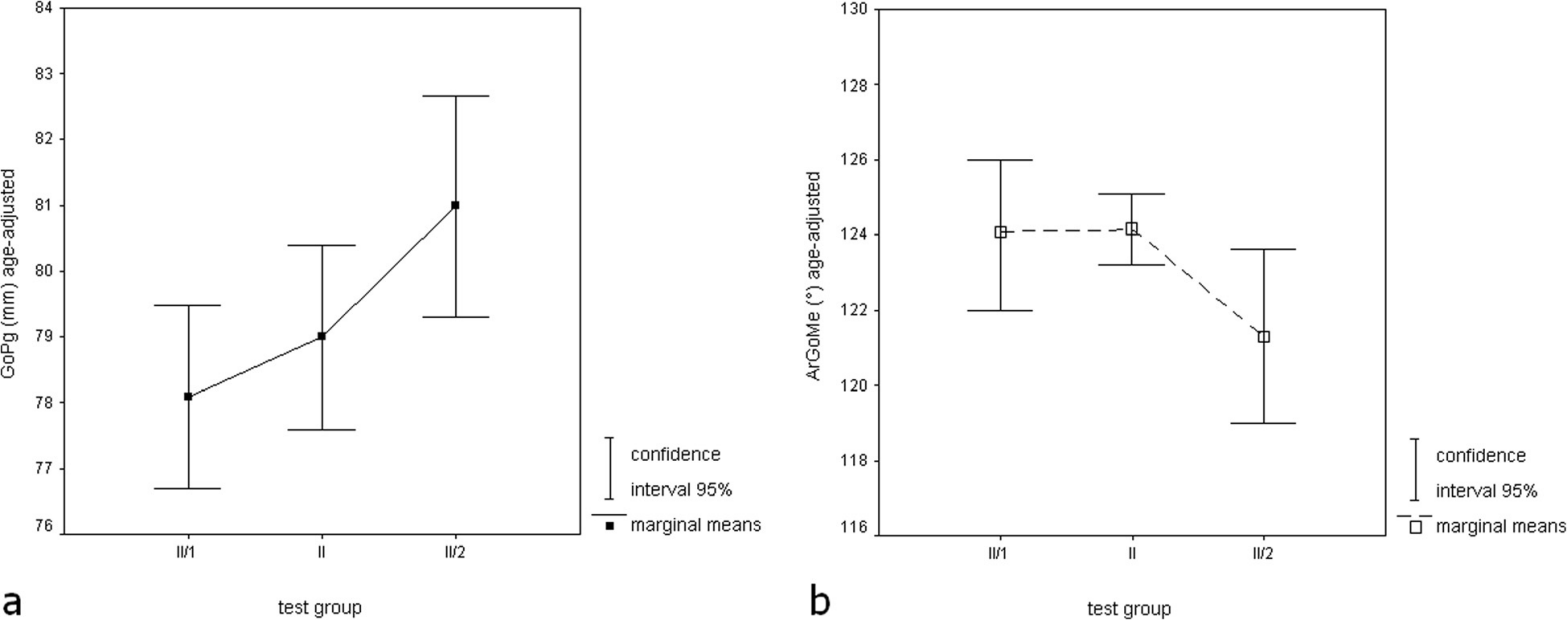
Age-adjusted group means (■) and 95 percent confidence intervals (□): a - mandibular total length (GoPg in millimeters); b - gonial angle (ArGoMe in degrees).

Sagittal parameters: The differences in maxillary length were not significant. In contrast, mandibular corpus and total lengths (Figure 2a) were significantly larger in group II/2 (75.4 and 81 mm respectively) compared with groups II and II/1 (p_τ_ < 0.001). No significant differences between groups were found for SNA, SNB, ANB, SNPg and PgNB angles, while Wits appraisal was smaller in group II/2 (1.5 mm) compared with group II (3.0 mm) and group II/1 (3.2 mm). This difference was significant (p = 0.01).

Vertical parameters: The height of the ascending ramus showed a trend toward reduction in group II/2 (p_τ_ = 0.05). The interbase angle ML-NL was not significantly larger in group II (25.1°) compared with groups II/1 (22.9°) and II/2 (22.1°). Gonial angle (Figure 2b) was found to decrease from groups II/1 (124.1°) and II (124.2°) to group II/2 (121.3°). This difference was significant (p_τ_ = 0.04).

Dentoalveolar parameters: Lower incisor inclination decreased from group II/1 to group II/2 (p_τ_ ≤ 0.003), while the interincisal angle increased (p_τ_ < 0.001) from group II/1 to group II/2. The differences in lower incisor inclination evaluated at L1-NB (degrees and mm) and interincisal angle were larger between groups II and II/2 than between groups II/1 and II.

Soft tissue parameters: The nasolabial angle increased from group II/1 (110.2°) to group II/2 (114°). The difference was significant (p_τ_ = 0.04).

## Discussion

The present study aimed to examine whether the *Angle* Class II subdivisions feature different dentoskeletal configurations. While some studies support the existence of pathognomonic skeletal features in Class II division 2 subjects clearly distinguishable from Class II division 1 morphology [2-4], others failed to ascertain in a larger sample fundamental skeletal differences between the two subdivisions [1]. These inconsistent findings may partly be due to a variable definition of malocclusions and insufficient control of confounding factors. To avoid such shortcomings, the present study relied on upper incisor inclination as the unique criterion to distinguish Class II subgroups ranked by decreasing proclination. However, improved interpretability of results is achieved at the cost of some detachment from common clinical understanding of the Class II entity which usually comprises a deep bite.

Patient age, gender and presence of a distal basal relationship were tested for confounding with the dentoskeletal configuration. While the distributions of gender and basal relationship were independent of group membership, the mean age of group II/2 patients was significantly higher by about 1.5 years than in the two other groups. Since age-matched test groups of sufficient sample size were not available, analyses were based on statistically estimated parameter values. Our ongoing unpublished research involving validation analyses on age subsets has proved the validity of this approach and yielded results consistent with those from statistical age adjustment. The significant findings presented, thus, are likely to be observed in Class II patients throughout the age span investigated. The multivariate approach has long been advocated by medical statisticians [15-17,19,20], but due to its abstract concept has not been widely used in clinical orthodontic investigations [21-23].

Lower incisor proclination significantly decreased from groups II/1 and II toward group II/2. A more retroclined position of the lower central incisors in Class II division 2 had been reported [24-27], also compared with division 1 [1,4,28,29]. The interincisal angle was found to increase continuously from group II/1 to group II/2 which agrees with other studies [4,30-33]. The magnitude of the interincisal angle is associated with the extent of vertical overbite, particularly in Class II division 2 patients [34-38].

In agreement with previous reports [2,4,39] maxillary length was not significantly different between the groups. Mandibular total and body length increased from group II/1 to group II/2 by about 3 mm on average, while results from literature are conflicting [2,3,40].

The present study revealed no significant differences in jaw position and basal relationship between the groups. Similarly, several previous studies failed to find clear differences in the basal positions between the Angle Class II subdivisions [1,2,4,28]. Some authors, however, reported a more retrognathic mandible [2,3,5,26,33,41,42] and a more obtuse ANB [3,28] in Class II division 1 as compared with division 2. Since ANB is largely affected by jaw prognathism and mandibular rotation, assessment of the anteroposterior basal relationship was additionally based upon Wits appraisal [14,43]. Statistical analysis revealed that the marginal means of group II/2 were only about half as high as in groups II/1 and II. *Brezniak*[4] also reported significantly smaller Wits values in division 2 patients as compared with division 1.

The marked chin prominence observed in Angle Class II division 2 [31,33] was not found to be different from division 1 in the present study. *Pancherz* and *Zieber*[32] who analyzed early and late mixed dentitions separately also could not show any differences .

Previous reports comparing Class II subdivisions [2-4] suggest that the more marked chin prominence of Class II division 2 patients develops not before the late mixed dentition, possibly due to increased growth inhibition of the alveolar process [7,44].

In agreement with earlier studies [1,2,28,30,32], no systematic covariation was found between the inclination of the upper incisors and that of either the maxilla or the mandible. In contrast, other authors reported a more acute mandibular plane angle in Class II division 2 as compared with division 1 [2-4,31]. A decreased mandibular plane angle may indicate anterior rotation of the mandible [27,45-47] resulting in a deep bite in Class II division 2 patients [4].

Most previous studies have found a variably reduced vertical interbase angle ML-NL in *Angle* Class II division 2 as compared with division 1 [1,2,4,5]. Correcting for age, the interbase angle appeared to be decreased by only about 1 mm in group II/2. Anterior rotation of the mandible may, however, be camouflaged by the remodelling processes occurring at the lower border [46,48,49] leading to physiological mandibular plane and interbase angles.

In summary, the craniofacial pattern of Class II division 2 subjects is more hypodivergent than in Class II division 1 [1,33,41]. Interestingly, patients with a normal incisor inclination (group II) showed a larger interbase angle compared with both other groups. This finding is not in conflict with the suggestion that anterior mandibular rotation is associated with a lack of incisor support, while a normal incisor inclination may safeguard against such rotation [31,50].

Reduction of the gonial angle is another indicator of a horizontal growth pattern with anterior mandibular rotation [46,51]. In agreement with previous findings [2,4] group II/2 showed a smaller gonial angle compared with the other groups. The existence of a mechanism compensating for an anterior position of the glenoid fossa was suggested to account for a smaller gonial angle in Class II division 2 patients [31,52].

A reduced lower anterior face height has frequently been described for Angle Class II [1,5,24,31,32,40,46,53]. However, the present study failed to reveal a significant association with incisor group membership. *Jarabak*’s ratio was also not a discriminating factor.

Due to the retroclined maxillary incisors, prominence of the upper lip was reduced in Class II division 2 compared with division 1. Consequently, the nasolabial angle was found to increase from group II/1 to group II/2. A further indication of soft tissue effects was obtained from the *Holdaway* angle which was slightly smaller in group II/2 patients compared with the other groups. Similar findings were reported by Isik *et al.*[3].

## Conclusions

The results of the present study show that before the permanent dentition is completed,

1 the systematic increase of the interincisal angle from Class II division 1 to Class II division 2 is due to decreased proclination also of the lower incisors,

2 the dimension and the anteroposterior position of the maxilla are not significantly different between incisor inclination subsets of Class II patients,

3 while the position of the mandible is not associated with incisor inclination itself, the Wits appraisal shows the distal basal relationship to decrease from proclined to retroclined Class II incisor position,

4 the increase of mandibular body length with decreasing incisor proclination observed in the present study requires further confirmation,

5 Class II inclination subgroups are not different for chin prominence,

6 upper lip prominence is reduced with decreasing incisor prominence.

## Consent

Written informed consent was obtained from the patient's guardian/parent/next of kin for the publication of this report and any accompanying images.

## Competing interests

The authors declare that they have no competing interests.

## Authors’ contributions

CK, PR, PP and CL contributed to the conception, design and coordination of the study. CK made substantial contributions to the acquisition of data and the preparation of the manuscript. CK drafted and wrote the manuscript. PR, PP and CL revised the manuscript. All authors read and approved the final manuscript.
